# How to get a bloodless intra-cardiac field in mini-invasive cardiac surgery

**DOI:** 10.1186/1749-8090-10-S1-A123

**Published:** 2015-12-16

**Authors:** Ludwig K von Segesser, Denis Berdajs, Saad AbdelSayed, Piergiorgio Tozzi, Enrico Ferrari

**Affiliations:** 1 CHUV, Lausanne, Switzerland; 2 Toronto General Hospital, Lausanne, ON, Canada

## Background/Introduction

Minimally-invasive cardiac surgery is increasingly used in conjunction with peripheral cannulation. However, due to the relatively small diameter of the access vessels, thin walled venous cannulas are usually used in conjunction with augmentation. We have previously reported about 36F self-expanding cannulas which allow for remote cannulation and full flow (2.4 l/min m2) by gravity drainage. The new, smaller virtually wall-less 24F cannulas which pass through an 18F percutaneous orifice were designed for augmented venous drainage.

## Aims/Objectives

The present study was designed to assess the in vivo performance of these, smaller, virtually wall-less, venous cannulas in comparison to traditional thin-wall cannulas.

## Method

Remote cannulation was realized in 4 porcine experiments (75 ± 3 kg) with percutaneous venous access, serial dilation up to 18F and insertion of either 19F thin wall, wire wound cannula or a smaller, virtually wall-less (24F Smartcanula ST). A standard MECC pump set with a centrifugal pump and a hollow fiber membrane oxygenator, but no in-line reservoir was used. Pump flow and the required pump inlet pressure were recorded for increasing pump speed from 1500 RPM to 3500 RPM (500 RPM increments).

## Results

Pump flow accounted for 1.2 ± 0.2 l/min for wall-less versus 1.2 ± 0.2 l/min for thin wall at 1500 RPM, 3.5 ± 0.4 versus 3.1 ± 0.4 at 2500 RPM, 5.6 ± 0.4 versus 4.2 ± 0.7 at 3500 RPM. Pump inlet pressure accounted for -4 ± 19 mmHg versus 2 ± 13 mmHg for 1500 RPM, -35 ± 19 versus -89 ± 27 at 2500 RPM, and -90 ± 21 versus -220 ± 29 for 3500 RPM. For a pump inlet pressure of -88 mmHg, the mean pump flow was 4.5 ± 0.8 l/min for the new smaller virtually wall-less venous cannula versus 3.1 ± l/min for control (p < 0.05).

## Discussion/Conclusion

At the well accepted pump inlet pressure of -80 mmHg, the new, smaller, virtually wall-less, braided cannulas designed for use with augmentation provide unmatched venous drainage in vivo despite an 18F peripheral access. Early clinical analyses have confirmed these findings.

